# Volumetric Radiological Analysis of Bone Regeneration After Jaw Cyst Enucleation Without Grafting Material: A Retrospective Pilot Study

**DOI:** 10.7759/cureus.93636

**Published:** 2025-10-01

**Authors:** Maria Sabbagh, Carla Maria Khairallah, Adam Saleh, Claude Chaanine

**Affiliations:** 1 Department of Oral Surgery, Saint Joseph University of Beirut, Beirut, LBN; 2 Department of Periodontology, Saint Joseph University of Beirut, Beirut, LBN

**Keywords:** enucleation, jaw cyst, shrinkage rate, three-dimensional imaging, volumetric analysis

## Abstract

Introduction

A jaw cyst is a pathological lesion that can affect both the maxillary and mandibular bones. The treatment of choice is complete surgical removal by enucleation. The healing process after enucleation has often been assessed using two-dimensional methods, but a three-dimensional approach may offer a more accurate evaluation. This study aims to assess whether the preoperative volume of a cyst influences the shrinkage rate after enucleation. It also explores the impact of other variables, such as age, gender, location, and defect configuration, on the shrinkage rate.

Methods

Preoperative and postoperative (after six to 12 months) cone beam computed tomography (CBCT) scans were collected from the Saint Joseph University of Beirut’s database (Beirut, Lebanon). Using the 3D Slicer® software, the scans were superimposed and then using segmentation, both preoperative and postoperative defects were isolated from other structures, and their volume was determined by the software. The shrinkage rate was then calculated.

Results

Twelve patients were selected from the database. The significance threshold was set at 0.05. Statistical analysis revealed no significant correlation between the shrinkage rate and preoperative volume (p > 0.05), age (p > 0.05), gender (p > 0.05), defect location (p > 0.05), or defect configuration (p > 0.05). However, a positive correlation was observed between the preoperative and the postoperative volume (p < 0.001).

Conclusion

While the results did not show a significant correlation between the different parameters and the shrinkage rate, the positive correlation between the preoperative and postoperative volume justifies the lack of correlation between the preoperative volume and the shrinkage rate.

## Introduction

A jaw cyst is a pathological entity that affects the maxillary and mandibular bones [1]. It typically appears as a radiolucent area on radiographic imaging [2,3]. The World Health Organization classifies these cystic lesions into two broad categories: odontogenic cysts of inflammatory origin and developmental cysts, which may be odontogenic or non-odontogenic [4].

Multiple radiological techniques can be used to visualize cystic lesions. Conventional panoramic radiographs are widely accessible and expose patients to a relatively low radiation dose. In many cases, this two-dimensional (2D) imaging modality is sufficient to support a differential diagnosis. However, it is important to remember that panoramic radiographs represent a 2D projection of a three-dimensional (3D) anatomical structure [5]. This limitation highlights the importance of cone beam computed tomography (CBCT). CBCT imaging has been shown to aid in lesion diagnosis, accurate localization, and surgical planning for cyst removal [6].

Various surgical approaches are available to treat jaw cysts. Marsupialization and decompression are conservative techniques that reduce cyst volume, thereby minimizing surgical risks and the extent of intervention [7-10]. Enucleation, on the other hand, is a more radical surgical technique that involves the complete removal of the cystic lesion [11]. According to the literature, enucleation remains the treatment of choice in most cases [1,3].

Although some studies have explored the use of bone grafting materials following enucleation, spontaneous bone regeneration is often achievable [2]. In fact, bone defects can heal naturally if the physiological healing sequence is preserved. First, a good blood supply forms a clot and fills the defect. Then, osteogenic and angiogenic cells migrate from the bone walls and periosteum, transforming the blood clot into granulation tissue, and subsequently into woven bone. Finally, the woven bone matures into a hard callus, which is remodeled over time by the coordinated actions of osteoclasts and osteoblasts [12,13].

The bone healing process can be monitored radiographically. On 2D images, assessments can be made through linear measurements in vertical, horizontal, and diagonal planes, comparing preoperative and postoperative radiographs [14]. However, a more accurate assessment of bone formation requires three-dimensional volumetric analysis [14]. This involves segmentation of the cystic defect on CBCT scans - a process by which the lesion is isolated from surrounding structures to generate a 3D model - allowing for precise calculation of the defect's volume [15].

The present study aims to investigate whether the initial size of a cystic lesion affects the shrinkage rate of the defect after enucleation without the use of bone grafting materials. Using a volumetric analysis approach based on preoperative and postoperative CBCT scans, the primary objective is to evaluate spontaneous bone healing after enucleation and assess whether the initial lesion’s size influences the shrinkage rate. In addition, the study considers other patient-related variables, including age, gender, lesion location, and defect configuration, to evaluate their impact on healing outcomes.

## Materials and methods

This retrospective cohort study was conducted in the Department of Oral and Maxillofacial Surgery at Saint Joseph University of Beirut (USJ) (Beirut, Lebanon), in collaboration with the Digital Dentistry Department and the Craniofacial Imaging Research Laboratory. Ethical approval was obtained from the university’s ethics committee on December 10, 2024 (approval no. Tfemd-2025-48). Patient data were retrieved from the university’s archives. The study included all patients who underwent enucleation of jaw cysts between January 2014 and February 2025 and had both preoperative and postoperative CBCT scans. Ninety-four files of patients who underwent jaw cyst treatments were screened. Only 12 patients met the inclusion criteria.

The inclusion criteria were as follows: presence of a cystic lesion in the maxilla or mandible treated by enucleation without the use of bone grafting material, availability of preoperative and postoperative CBCT scans taken six to 12 months apart, and medically healthy status. The exclusion criteria were as follows: patients with uncontrolled systemic diseases, patients taking any medication that may interfere with bone formation, cystic lesions treated by marsupialization, cystic cavities filled with any type of bone graft material, and recurrent cysts.

A total of 12 patients met the inclusion criteria. Data collected included patients’ age, gender, lesion location, and duration of follow-up. Volumetric measurements of the cystic lesions were performed on the preoperative (T0) and postoperative (T1) CBCT scans.

The scans were exported in Digital Imaging and Communications in Medicine (DICOM) format and imported into the 3D Slicer® software. An initial alignment was performed using the fiducial registration module, which employs a landmark-based superimposition technique by selecting three or more common anatomical landmarks on both scans. Final alignment was achieved using the “BRAINS” general registration module, a voxel-based superimposition method.

**Figure 1:**
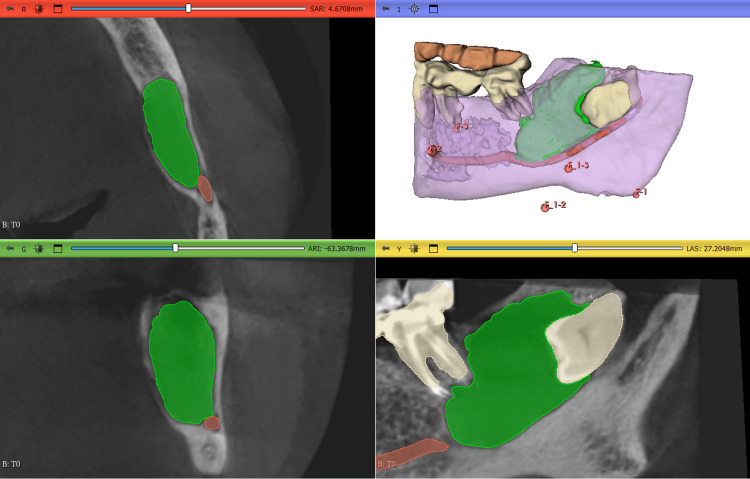
Segmentation and 3D model of the T0 scan

On the T0 scan, automatic segmentation of radiopaque structures such as the maxilla or mandible, teeth, and the mandibular canal was performed using the “Dental Segmentator” extension. The bone defect, corresponding to the cystic lesion, appeared as a radiolucent area and was therefore left unsegmented. Manual segmentation of the cystic lesion was performed using a thresholding method and the sphere brush tool, isolating the defect outside all previously identified structures. The resulting segmentation of the T0 defect was converted into a 3D model, and the volume was calculated and displayed in the “Models” module (Fig. 1).

**Figure 2:**
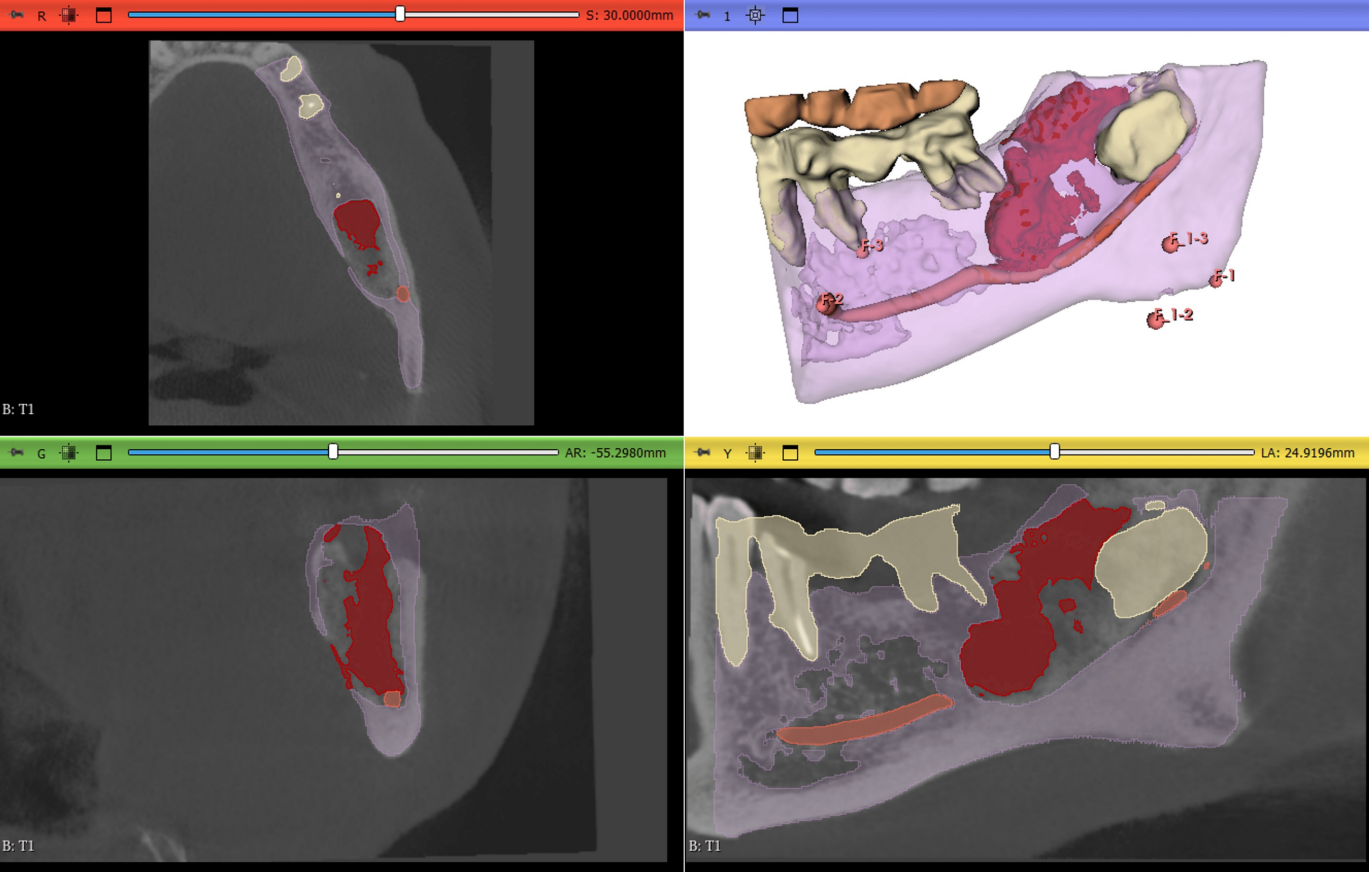
Segmentation and 3D model of the T1 scan

On the T1 scan, the threshold was similarly determined, and a manual segmentation was performed within the boundaries of the T0 defect segmentation. This allowed for assessment of the remaining bone defect at follow-up. The T1 segmentation was also converted into a model, and the volume was calculated and displayed in the “Models” module (Fig. 2).

Shrinkage rate formula

Based on the article by Ku et al. (2022) [2], the shrinkage rate was calculated following this formula:

Shrinkage rate (%) = [(Preoperative volume (mm³) − Follow-up volume (mm³)) / Preoperative volume (mm³)] × 100

Statistical analysis was performed using IBM SPSS Statistics for Windows®, Version 25.0 (released 2017, IBM Corp., Armonk, NY). The significance threshold was set at 0.05. The statistical tests used in this study were the Mann-Whitney U-test or Student’s t-test (evaluated by the Shapiro-Wilk test), Pearson’s correlation test, and Kruskal-Wallis or ANOVA test (evaluated by the Shapiro-Wilk test).

## Results

The population consisted of 12 patients, five of whom were female patients with an age of 39.2 ± 19.4 years and seven were male patients with an age of 55.9 ± 6.9 years. There was no statistically significant difference in age between the two genders (p > 0.05).

Overall, the mean preoperative volume of the cyst was 1529.4 ± 1096.3 mm^3^ with residual defects of 904.2 ± 771.2 mm^3^ and a shrinking rate of 43.2 ± 14.1% at 9 ± 2.5 months follow-up.

Relationship between preoperative volume and shrinking rate

**Figure 3:**
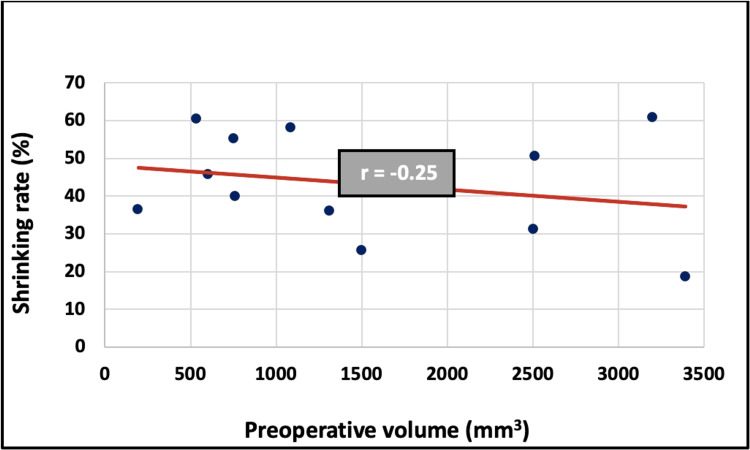
Correlation between preoperative volume and shrinking rate

Pearson’s correlation test did not reveal any statistically significant correlation between preoperative volume and shrinking rate (r = -0.25, p > 0.05, Fig. 3).

Relationship between preoperative and follow-up volumes

**Figure 4:**
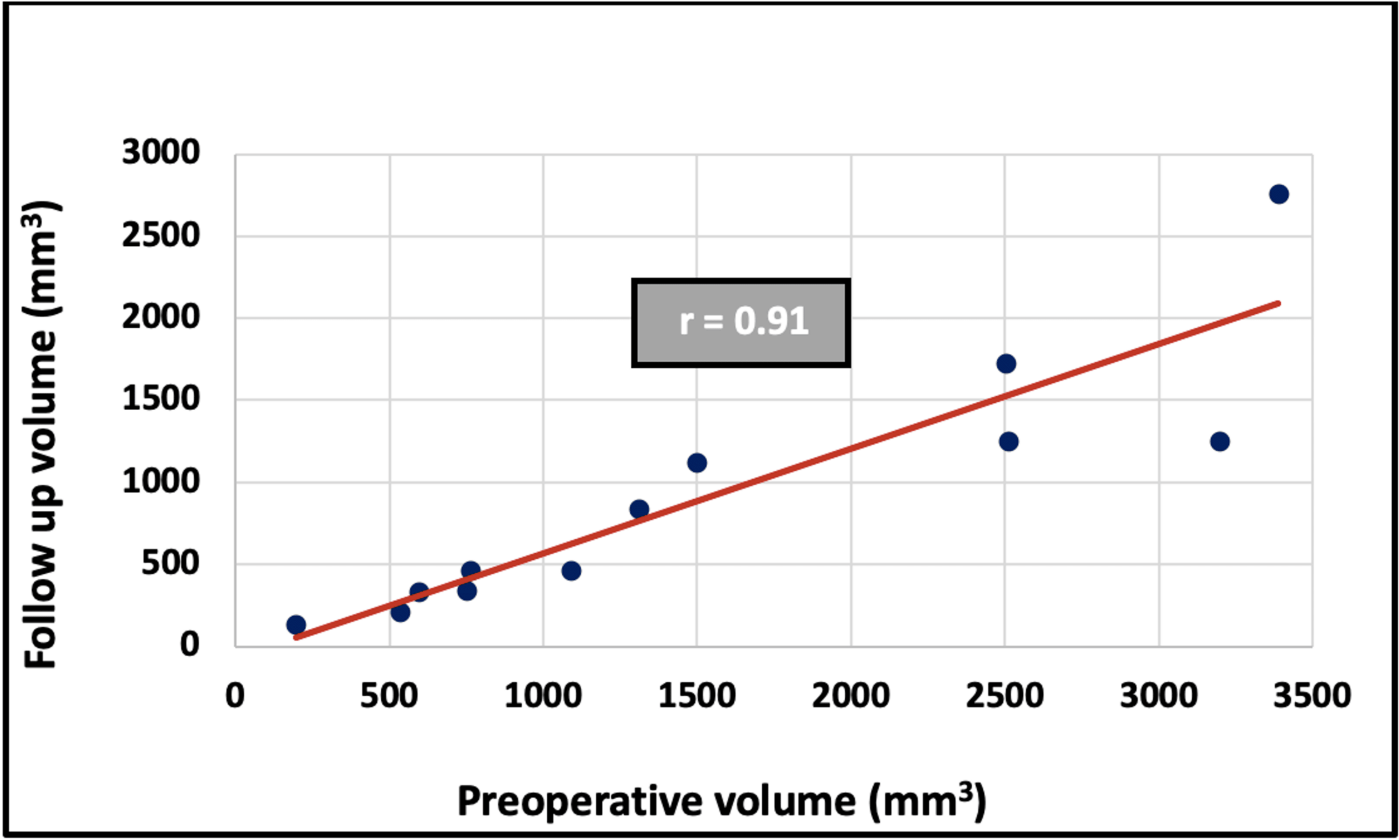
Correlation between preoperative volume and follow-up volume

Pearson’s correlation test revealed that follow-up volume was positively correlated to preoperative volume (r = 0.91, p < 0.001, Fig. 4).

Relationship between age and shrinking rate

**Figure 5:**
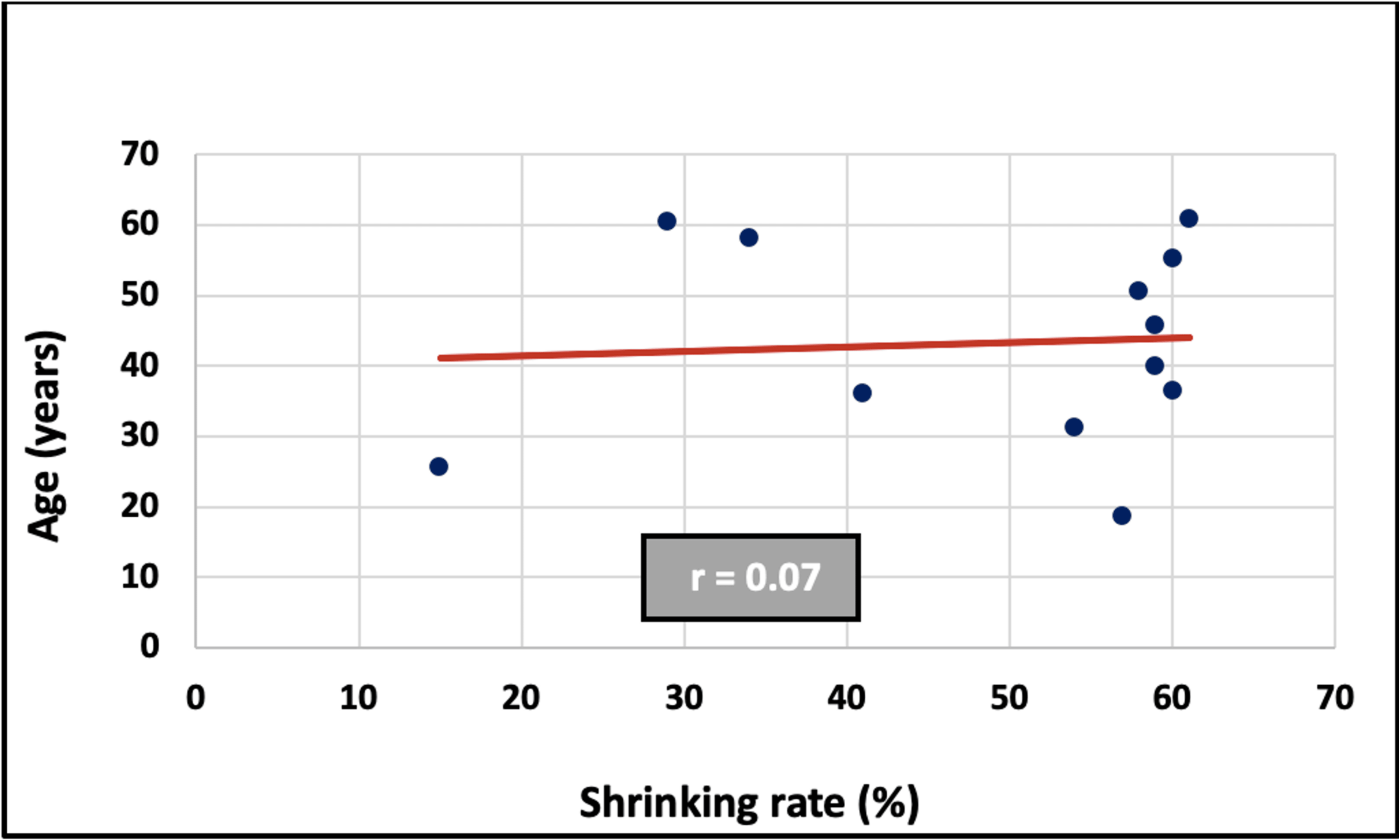
Correlation between age and shrinking rate

Pearson’s correlation test did not reveal any statistically significant correlation between age and shrinking rate (r = 0.07, p > 0.05, Fig. 5).

Relationship between shrinking rate and gender groups

**Figure 6:**
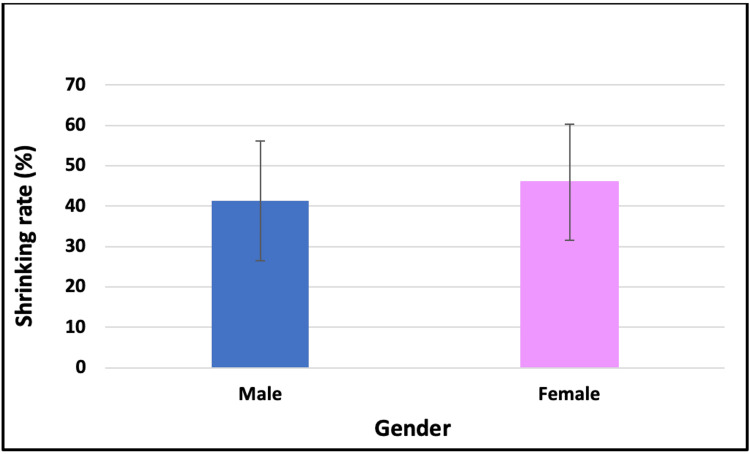
Relationship between shrinking rate and gender categories

There was no statistically significant difference in the shrinking rate between the male and female groups (41.3 ± 14.8 vs. 45.9 ± 14.3 resp., p > 0.05, Fig. 6). 

Relationship between shrinking rate and location categories

**Figure 7:**
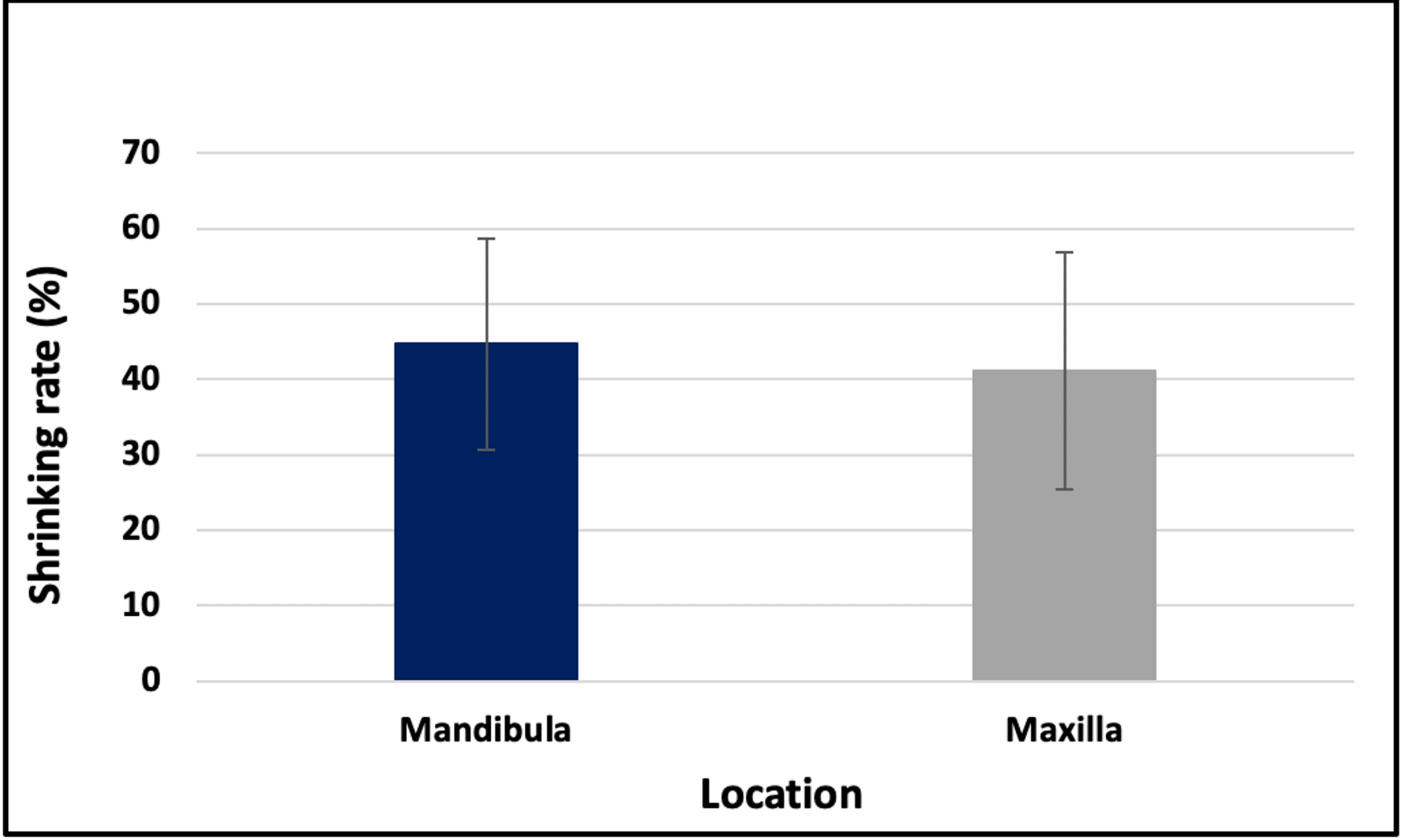
Relationship between shrinking rate and location categories

There was no statistically significant difference in the shrinking rate between the mandibular and maxillary location groups (44.7 ± 13.9 vs. 41.2 ± 15.7 resp., p > 0.05, Fig. 7).

Relationship between shrinking rate and the defect configuration (the presence or absence of a buccal or palatal/lingual wall)

**Figure 8:**
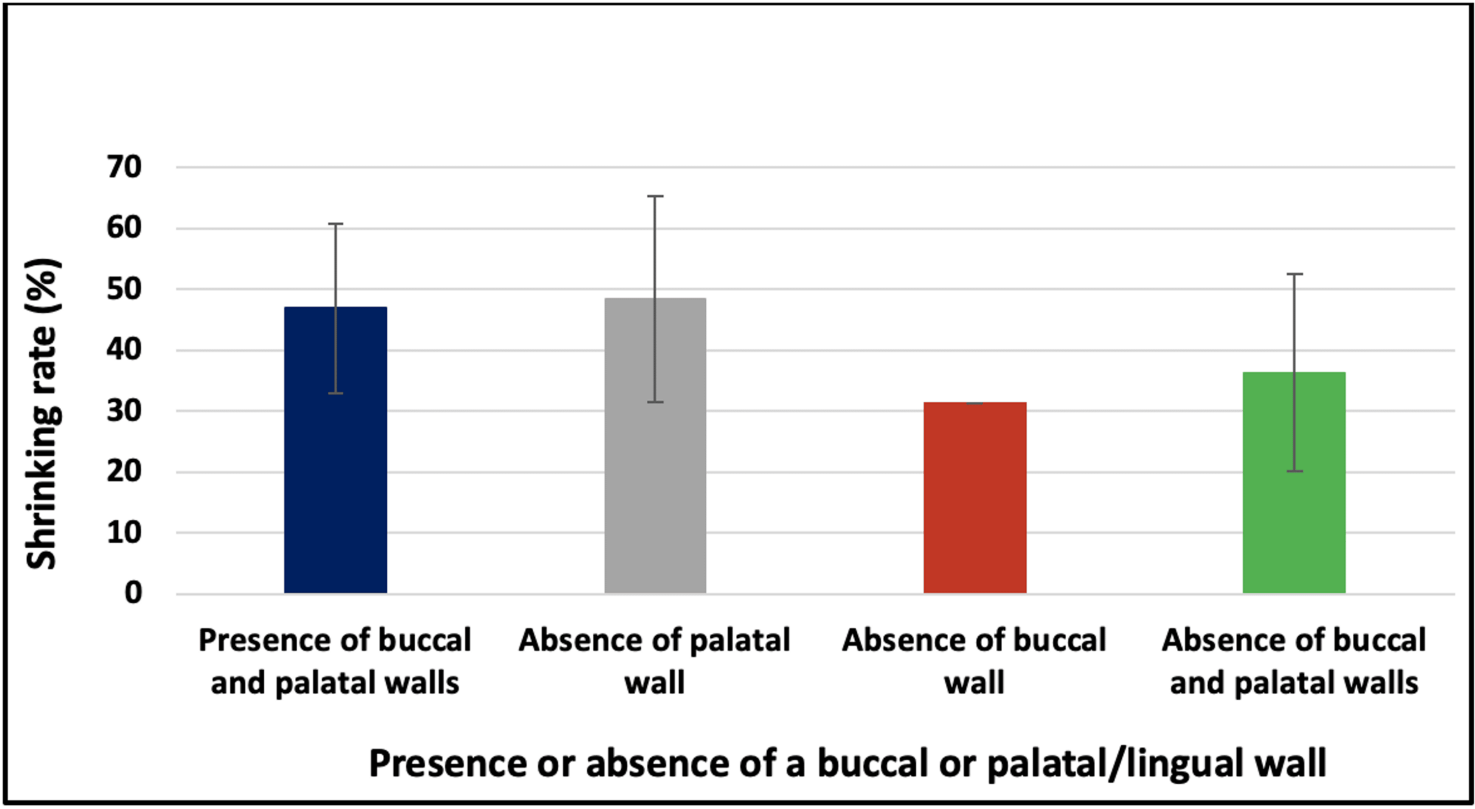
Relationship between shrinking rate and defect configuration (the presence or absence of a buccal or palatal/lingual wall)

There was no statistically significant difference in the shrinking rate between the presence of buccal and palatal walls, the absence of palatal wall, the absence of buccal wall, and the absence of the buccal and palatal wall groups (46.9 ± 13.9 vs. 48.4 ± 16.9 vs. 31.3 ± 0 vs. 36.3 ± 16.2 resp., p > 0.05, Fig. 8).

## Discussion

The primary objective of this study was to evaluate whether the preoperative volume of a cystic lesion influences the shrinkage rate of the residual bone defect after enucleation without the use of bone grafting material. Secondary variables such as age, gender, defect location, and defect configuration (presence or absence of buccal and palatal/lingual walls) were also assessed for their potential impact on shrinkage rate. Volumetric analysis was conducted using the 3D Slicer® software.

Statistical analysis revealed a strong positive correlation between the preoperative volume (T0) and the follow-up volume (T1), with a correlation coefficient of r = 0.91. This suggests that T1 increases proportionally with T0. However, no statistically significant correlation was observed between preoperative volume and shrinkage rate. Since the shrinkage rate formula is based on both T0 and T1, and given their strong linear relationship, the shrinkage rate tends to remain constant regardless of the initial lesion size. This implies that larger lesions leave behind proportionally larger defects, but the percentage of volume reduction remains constant. This explains the lack of statistical significance between T0 and the shrinkage rate

These findings are consistent with prior research. Rubio et al. (2015), using 2D radiographic analysis, reported that initial cavity size did not significantly affect bone formation [14]. Similarly, Ku et al. (2022) found no correlation between preoperative volume and healing ratio in a 3D volumetric analysis and confirmed that larger cysts leave larger defects [2]. However, Al-Qurmoti et al. (2023) suggested that smaller lesions showed a greater percentage of volume reduction, indicating a potential inverse relationship, which is in disagreement with the current results [3]. In addition, Rosa et al. (2024) reported a negative correlation between preoperative volume and shrinkage rate, concluding that larger lesions had a lower shrinkage rate [16]. 

Regarding the patient’s age, the results showed no significant correlation with the shrinkage rate. This finding does not align with the results of Al-Qurmoti et al. (2023), who reported higher shrinkage rates in younger patients [3]. Ku et al. (2022) similarly reported that age was not a significant factor during the first year of follow-up, but became significant after one year, indicating that younger individuals demonstrated higher healing ratios in longer follow-up durations [2]. These discrepancies may be attributed to the small sample size (n=12) and the limited follow-up period in the present study.

In terms of gender, no statistically significant difference was observed, although females demonstrated a slightly higher mean shrinkage rate (45.9 ± 14.3) compared to males (41.3 ± 14.8). This aligns with one study reporting smaller residual defects in females [3]. Another study showed that there was not a statistically significant difference in the healing ratio between males and females [2]. Research has shown that females tend to have slower bone regeneration, and that may be due to hormonal differences [3]. These conflicting findings highlight the need for further research for a better understanding of the effect of gender on the shrinkage rate.

As for defect location, the present study showed no statistically significant difference in the shrinkage rate between the maxilla and the mandible. Although the mean shrinkage was slightly higher in mandibular lesions (44.7 ± 13.9%) than maxillary ones (41.2 ± 15.7%). Previous studies using 2D radiographs indicated higher bone density increases in mandibular defects [17,18], whereas Ku et al. (2022) reported no significant difference in healing ratio between the jaws using 3D volumetric measurements [2]. These differences in results may be due to the methodology of the analysis of the healing process. While densitometry on 2D radiographs can be an effective tool to evaluate bone regeneration [19], Buchbender et al. (2020) implied that 3D volumetric measurements offer a more precise evaluation of bone healing. That study also found no statistically significant difference in defect healing based on location [20].

The literature suggests that the presence of buccal and palatal/lingual walls facilitates blood clot stabilization and, therefore, enhances bone healing. It is also believed that the rupture of either cortical plate may lead to fibrous scaring and incomplete osseous fill [14,20]. However, the present study did not find a significant difference in the shrinkage rate between different defect configurations. This agrees with the results of the study conducted by Buchbender et al. (2020), which found no significant difference between the defects’ configuration. Nevertheless, it was implied that this parameter needs more investigation, especially using 3D analysis [20].

This study has some limitations. Most importantly, the small sample size (n = 12), which limits the generalizability of the findings and reduces statistical power, possibly contributes to the lack of significance in some comparisons. Furthermore, the follow-up duration was restricted to six to 12 months, which may not fully capture the longer-term bone remodeling process. Future research involving larger patient cohorts and longer follow-up periods is essential to validate these preliminary findings and explore the clinical implications of lesion volume and other parameters on bone regeneration.

## Conclusions

The findings of this study indicate that the shrinkage rate of bone defects following jaw cyst enucleation without the use of bone grafting material was not significantly influenced by the preoperative volume, patient age, gender, defect location, or defect configuration. However, a strong positive correlation was observed between the preoperative and follow-up volumes, which explains the absence of a statistically significant relationship between the preoperative volume and the shrinkage rate, as the latter tends to remain relatively constant regardless of lesion size. Further research is warranted with a larger sample size and additional follow-up scans to gain a deeper understanding of the healing process and defect shrinkage over time.
